# *Cyclo*‐N_9_^−^: a Novel 5/6 Fused Polynitrogen Anion for High Energy Density Materials

**DOI:** 10.1002/advs.202414394

**Published:** 2025-01-28

**Authors:** Xiaofeng Yuan, Ze Xu, Haolin Gu, Tongwei Zhang, Yuangang Xu, Ming Lu

**Affiliations:** ^1^ School of Chemistry and Chemical Engineering Nanjing University of Science and Technology Nanjing Jiangsu 210094 China

**Keywords:** *cyclo*‐N_9_^−^, detonation performance, ionic interactions, polynitrogen anion, quantum calculations

## Abstract

After *cyclo*‐pentazolate anion, a 5/6 fused structure of N_9_^−^ is constructed, and four novel nitrogen‐rich ionic compounds are assembled on its basis. The results of the quantum calculations revealed an uneven distribution of electrons on *cyclo*‐N_9_^−^, with significant charge density near the N5/N9 atoms and an ADCH charge of −0.425. The relative strength of chemical bonds is assessed through bond order analysis, which is further supplemented by transition state theory and ab initio molecular dynamics, ultimately leading to the identification of the decomposition pathways of *cyclo*‐N_9_^−^. The aromaticity of *cyclo*‐N_9_^−^ and its individual sub‐rings is cleverly validated through a combination of NICS_ZZ and ICSS methods. Among the eight systems, *cyclo*‐N_9_^−^ forms hydrogen bonds with other cations, and IGMH analysis revealed significant LP‐π and π–π stacking interactions between [N_7_H_4_^+^] and *cyclo*‐N_9_^−^, both of which enhance system stability. The theoretical energy densities in all systems are at the forefront in the currently emerging nitrogen‐rich compounds. Attributed to its extraordinarily high enthalpy of formation, the detonation performance of [N_7_H_4_^+^] [N_9_^−^] is particularly excellent. However, [NH_3_OH^+^] [N_9_^−^] exhibits better stability and most exciting performance, making it a highly promising candidate with application potential.

## Graphical abstract

This study presents a novel 5/6 fused polynitrogen anion, *cyclo*‐N_9_^−^, which is assembled with four cations to form energetic salts. The computational results reveal that the energy densities and detonation performances of these systems are far superior, even surpassing those of CL‐20. This study seeks to offer significant theoretical insights for the syntheses of polynitrogen compounds.

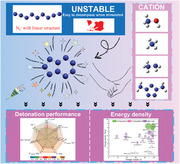

## 1 Introduction

Recent advancements in technology have resulted in more rigorous standards for weapons and ammunition on the battlefield. As a result, the emphasis on synthesizing energetic compounds has progressively transitioned from conventional CHON materials to high‐energy‐density materials (HEDMs), including nitrogen‐rich and polynitrogen compounds.^[^ ^1^, ^2^, ^3^, ^4^ ^]^ These materials primarily derive their energy from high enthalpy of formation, rather than from the oxidation of carbon frameworks inside the molecules. This allows them to explode in environments with low oxygen content, such as high altitudes.^[^ ^5^ ^]^ Moreover, nitrogen‐based structures like N‐N, N = N, and N≡N bonds can release astonishingly large amounts of energy, and their detonation products are mainly N₂, which is ecologically harmless.^[^ ^6^, ^7^ ^]^ The numerous advantages of these materials have made increasing the nitrogen content in explosives a key direction in the field of energetic materials, aiming to enhance detonation performance.^[^ ^8^ ^]^ In the mid‐20th century, Ugi completed the synthesis of aryl pentazole for the first time. However, these compounds could only exist at low temperatures, and the pentazole rings were highly prone to decomposition.^[^ ^9^ ^]^ Regrettably, in the subsequent years, the synthesis of polynitrogen compounds encountered an extended phase of standstill. In 1999, the American Air Force Research Laboratory (AFRL) synthesized the third polynitrogen species, the N₅⁺ cation. Nonetheless, its linear configuration obstructs electron delocalization and makes it lacked aromaticity, leading to decomposition at lower temperatures.^[^ ^10^ ^]^ Meanwhile, theoretical calculations of polynitrogen compounds were also in full swing.^[^ ^11^, ^12^ ^]^ Lein et al. conducted quantitative calculations on dicyclopentadienyliron at the B3LYP/6‐311+G* level, revealing that iron can effectively enhance the stability of N_5_^−^ with a binding energy of 109.0 kcal mol^−1^. This suggests that it could become a highly promising pentazole metal salt for synthesis.^[^ ^13^ ^]^ Tobita, on the other hand, investigated the stability and spectra of various isomers of N_6_. The study revealed that within a series of N_6_ isomers, the C_2v_ configuration of N_6_ exhibits the lowest energy, suggesting that N_6_ molecules conforming to this point group possess greater stability.^[^ ^14^ ^]^ Subsequently, in 2017, Oleynik and colleagues successfully synthesized CsN₅ crystals under pressures approaching 60 GPa. High‐pressure and high‐temperature experiments, guided by first‐principles crystal structure predictions, demonstrated that the crystal system of synthesized CsN₅ is consistent with the theoretical predictions results.^[^ ^15^ ^]^ In the same year, Zhang, building on the experimental work of Professor Haas,^[^ ^16^ ^]^ successfully prepared the world's first stable pentazolate salt (N₅)₆(H₃O)₃(NH₄)₄Cl at room temperature, with a decomposition temperature as high as 117 °C.^[^ ^17^ ^]^ Despite facing skepticism,^[^ ^18^, ^19^, ^20^ ^]^ Xu later succeeded in synthesizing a series of stable metal pentazolate salts, igniting excitement in the field of polynitrogen compound synthesis.^[^ ^21^ ^]^

**Figure 1:**
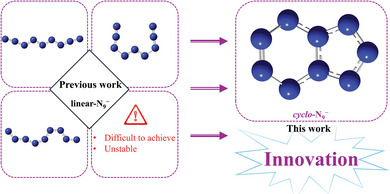
The comparison between the previous N_9_^−^ model and the innovative model.

Looking back at the progress made in increasing the nitrogen content in energetic materials, it appears that the transition from N_3_^−^ to N_5_^−^ underscores the significance of stable N_m_^−^ anions in the synthesis of polynitrogen compounds. This has naturally drawn attention to nitrogen clusters such as N₉⁻. In fact, theoretical studies on N₉⁻ clusters began quite early.^[^ ^22^ ^]^ However, the models constructed were seemingly influenced by the N₅⁺ cation, and most research on N₉⁻ was based on linear structures, whose stability was questionable.^[^ ^23^ ^]^ Moreover, obsolete technology and insufficient specialized expertise at that time resulted in many initial research outcomes failing to satisfy contemporary synthetic requirements. Motivated by the synthesis of K₉N₅₆ by Dominique Laniel and our successful detection of diamino‐pentazolium cation signals in MS,^[^ ^24^, ^25^ ^]^ we have refocused our efforts on *cyclo*‐N_9_^−^. Leveraging the reactive sites on diamino‐pentazolium, the preparation of *cyclo*‐N_9_^−^, a pentazole‐fused hexazine, appears highly promising. Therefore, in this study, based on quantum calculations, the *cyclo*‐N_9_^−^ anion with the *cyclo*‐N_5_ as the main ring was constructed, and its electronic structure and aromaticity were investigated and validated. The bond order analysis focused on discussing the strength of chemical bonds, while transition state theory and ab initio molecular dynamics (AIMD) were employed to explore the chemical bond stability of *cyclo*‐N_9_^−^. **Figure** **1** illustrates the contrast between the N_9_^−^ model developed in prior research and the model introduced in this study.

**Figure 2:**
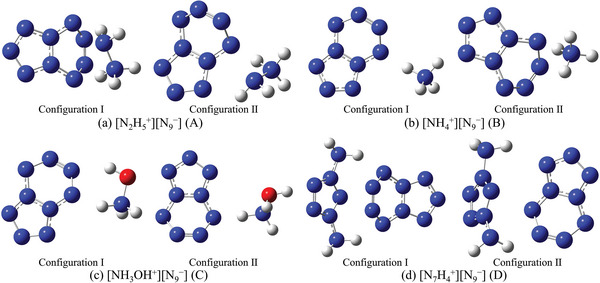
Structure diagrams of eight ionic systems.

To assess its practical utility, *cyclo*‐N_9_^−^ was assembled with cations to form four ionic compounds: [N_7_H_4_^+^] [N_9_^−^], [N_2_H_5_^+^] [N_9_^−^], [NH_4_^+^] [N_9_^−^], and [NH_3_OH^+^] [N_9_^−^]. Among them, N_2_H_5_^+^, NH_4_^+^ and NH_3_OH^+^ are common cations in energetic ionic salts. While the inclusion of N_7_H_4_^+^ is inspired by our previous work, which is expected to significantly enhance the detonation performance of the ionic systems. For each compound, two isomers were selected to investigate the variations arising from different configurations within the same ionic compound, resulting in a total of eight systems. Among these, configuration I is unequivocally the lowest‐energy structure across all configurations considered. configuration II, on the other hand, was chosen based on its position as the second lowest‐energy structure, with a significantly different atomic distribution compared to configuration I. As expected, the theoretical stability of configuration II is notably lower than that of configuration I. For simplicity and ease of discussion, the four ionic compounds are referred to as A, B, C, and D, respectively. The configurations of all compounds are illustrated in **Figure** **2**. The interactions between the cations and anions were analyzed using Atoms in Molecules (AIM),^[^ ^26^ ^]^ Electrostatic potential (ESP)^[^ ^27^ ^]^ and Independent gradient model based on Hirshfeld (IGMH),^[^ ^28^ ^]^ complemented by interaction energy calculations and symmetric‐adapted perturbation theory (SAPT).^[^ ^29^ ^]^ Ultimately, the detonation performance of the eight systems was calculated, with stability and energy being thoroughly considered. This study not only provides a comprehensive theoretical foundation for our future synthesis of polynitrogen compounds but also offers valuable insights and guidance for experimental work by other researchers.

## 2 Result and Discussion

### 2.1 Electronic Structure and Chemical Bond Stability of *cyclo*‐N_9_^−^

**Figure 3:**
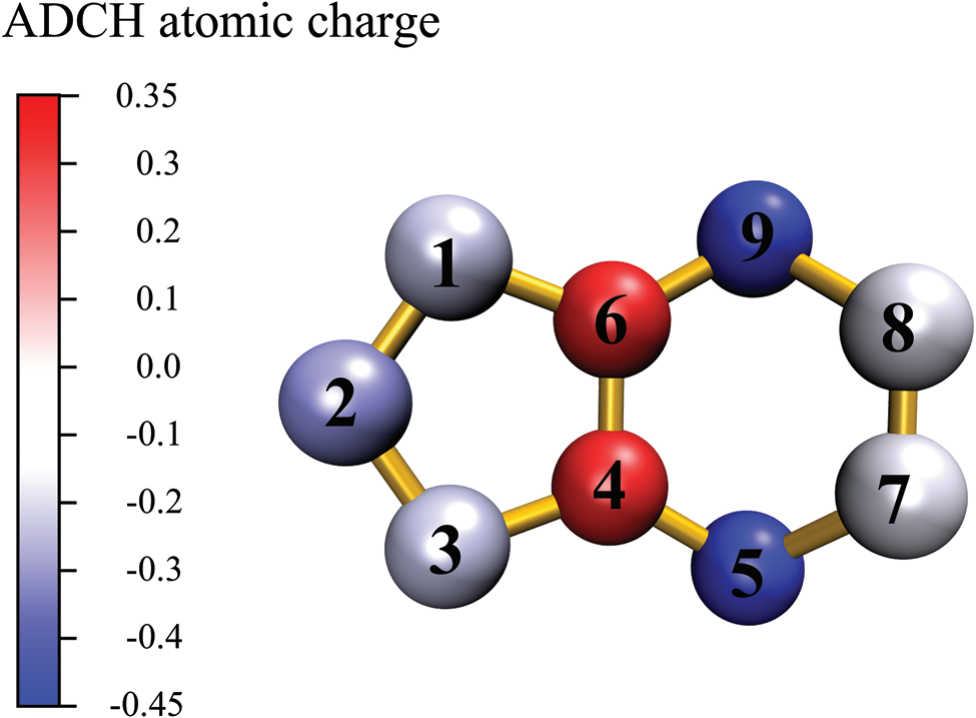
Atomic charge distribution of *cyclo*‐N_9_^−^ displayed by atomic coloring method.

Figure S1 (Supporting Information) shows the optimized structure of *cyclo*‐N_9_^−^. It is evident from the figure that the pentazole ring and the hexazine ring are not coplanar. This is primarily because the charge distribution among the nine nitrogen atoms in *cyclo*‐N_9_^−^ is not as uniform as in *cyclo*‐N_5_^−^, causing some nitrogen atoms to shift out of the plane to stabilize the entire ion. Atomic dipole moment corrected Hirshfeld (ADCH)^[^ ^30^ ^]^ expands all atomic dipole moments into correction charges based on the calculation of the Hirshfeld charge and atomic dipole moment of each atom. Unlike traditional Hirshfeld charges, which often have smaller values and poor reproducibility of dipole moments and electrostatic potentials, ADCH addresses these limitations effectively, providing a more reliable description of atomic charges. Thus, **Figure** **3** visually displays the distribution of charges on different atoms using the ADCH. With the exception of the positively charged nitrogen atoms at the junction, the other atoms carry negative charges distributed on both sides. Additionally, the darker color of the N5 and N9 atoms indicates a higher electron density in these regions. To provide a more concrete representation of the atomic charges in *cyclo*‐N_9_^−^, three different methods—ADCH, Natural Population Analysis (NPA),^[^ ^31^ ^]^ and Hirshfeld^[^ ^32^ ^]^—were used to calculate the charge values, which were then compared with the data for *cyclo*‐N_5_^−^ as shown in **Table** **1**. Although these methods are based on different theoretical calculations, they all reveal the same trend. First, the electron distribution in *cyclo*‐N_5_^−^ is uniform; the charge on each of the five nitrogen atoms is −0.2 across different methods, which contributes to the stability of the entire ring. However, the charge distribution in *cyclo*‐N_9_^−^ is not uniform. The negative charges on N5 and N9 are the smallest, with ADCH values of −0.425, indicating higher electron density at these positions. This makes them more likely to attract positive groups or molecular fragments to form hydrogen bonds or undergo chemical reactions.

**Table 1:** Atomic charge values calculated by three methods.

System	Atom	Atomic charge [ADCH]	Atomic charge [NPA]	Atomic charge [Hirshfeld]
*cyclo*‐N_5_^−^	N	−0.200	−0.200	−0.200
*cyclo*‐N_9_^−^	N1/ N3	−0.154	−0.058	−0.128
	N2	−0.233	−0.059	−0.128
	N4/ N6	0.313	0.074	0.107
	N5/ N9	−0.425	−0.206	−0.301
	N7/ N8	−0.117	−0.031	−0.115

Combining the data from Figure 3 and Table 1, it is fortunate that the charge distribution in *cyclo*‐N_9_^−^ exhibits symmetry. Except for the N2 atom, the remaining nitrogen atoms have pairs where the charge is identical, which may contribute to the overall stability of the system. To further explore the structure and properties of *cyclo*‐N_9_^−^, the bond lengths and bond orders of different chemical bonds were calculated. It should be noted that the common Mayer bond order is not compatible with diffuse functions and can even lead to erroneous results. Therefore, the analysis only includes Fuzzy bond order (FBO),^[^ ^33^ ^]^ Wiberg bond order based on Lowdin orthogonalization (WBO),^[^ ^34^ ^]^ and Laplacian bond order (LBO).^[^ ^35^ ^]^ The calculated bond orders are shown in Table S1 (Supporting Information).

**Figure 4:**
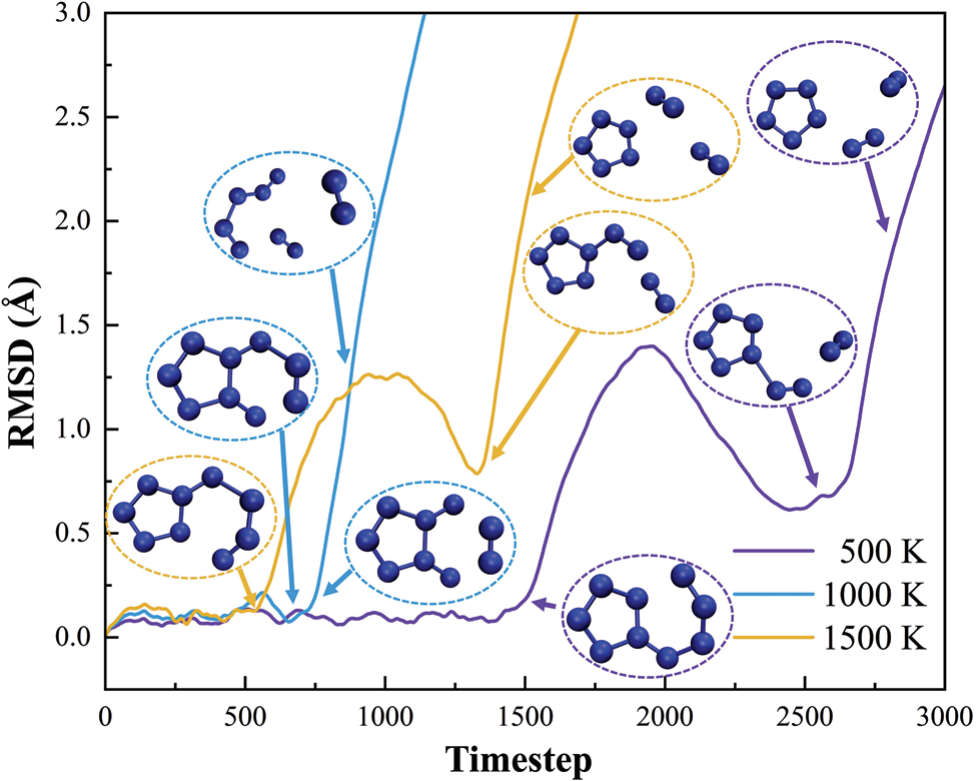
The RMSD variations and molecular snapshots of *cyclo*‐N_9_^−^ at different time points under various temperatures.

The variation in bond order is similar to the atomic charge distribution, also exhibiting a certain degree of symmetry. The bond formed between N7 and N8 has the highest bond order, followed by the N1‐N2 and N2‐N3 bonds, indicating these three bonds are more resistant to decomposition. Generally, there is a positive correlation between bond order and bond stability, suggesting that the N7‐N8 bond has the best stability. The results from all three methods indicate that the bond orders of N9‐N8 and N5‐N7 are lower, implying poorer stability.^[^ ^36^, ^37^ ^]^ In order to verify this view and better evaluate the stability of *cyclo*‐N_9_^−^, the AIMD calculations of *cyclo*‐N_9_^−^ were performed at 500, 1000, and 1500 K, and its root‐mean‐square deviation (RMSD) was statistically analyzed, as shown in **Figure** **4**. The structure of the first frame is used as a reference. When *cyclo*‐N_9_^−^ undergoes decomposition, the rapid change in atomic positions causes the RMSD curve to rise sharply, and the different configurations in the figure represent decomposition products at various time points.

**Figure 5:**
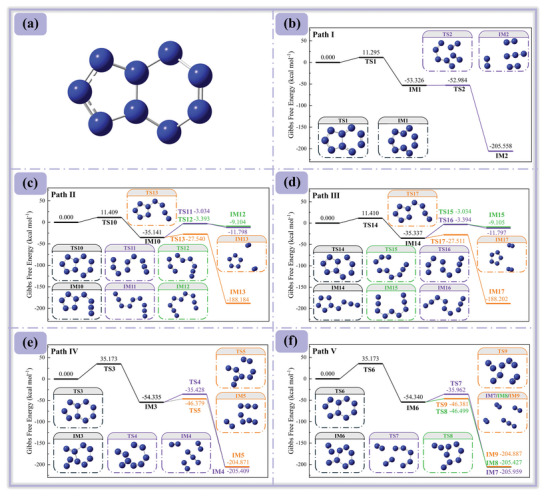
Decomposition mechanism and energy barrier of *cyclo*‐N_9_^−^. Among them, a) Initial configuration *cyclo*‐N_9_^−^; b), c) and d) indicate the ring‐opening positions along the reaction pathway at *cyclo*‐N_6_; e) and f) indicate the ring‐opening positions along the reaction pathway at *cyclo*‐N_5_.

At 1000 K, *cyclo*‐N_9_^−^ undergoes cleavage of the N5‐N7 and N8‐N9 bonds, resulting in the release of an N_2_ molecule. At 500 K and 1500 K, the initial cleavage occurs at either the N4‐N5 or N6‐N9 bonds, which is then followed by the cleavage of the N7‐N8 bonds, ultimately leading to the release of an N_2_ molecule. Interestingly, across all three temperatures, no ring‐opening reaction was observed for *cyclo*‐N_5_, even after 20 ps. These results provide dual support for our analysis. On the one hand, they corroborate the bond order analysis, which indicates that the N5‐N7, N8‐N9, N4‐N5, and N6‐N9 bonds have lower bond orders and, therefore, lower stability. On the other hand, the distinct reaction pathway at 1000 K compared to the other two temperatures suggests that *cyclo*‐N_9_^−^ involves multiple decomposition pathways. However, comparing the relative strengths of the N5‐N7/N8‐N9 bonds with the N4‐N5/N6‐N9 bonds cannot be fully achieved through AIMD alone. To address this, we calculated the Gibbs free energy barriers for the cleavage of different bonds using transition state theory, as shown in **Figure** **5**.

Figure 5 reveals two major decomposition pathways for *cyclo*‐N_9_^−^, determined by different ring‐opening positions: one leading to *cyclo*‐N_5_ and the other to *cyclo*‐N_6_. The transition state pathways depicted in Figure 5 not only encompass the configurations observed in AIMD simulations but also provide additional insights into the decomposition process of *cyclo*‐N_5_. For the *cyclo*‐N_6_ pathway, the energy barriers for the three transition states are nearly identical, at ≈11 kcal mol^−1^. This is comparable to the energy barriers observed in the initial decomposition pathways for N_8_ and N_10_, which is a common feature of polynitrogen compounds.^[^ ^8^, ^38^ ^]^ Numerically, the energy barriers for the cleavage of the N5‐N7/N8‐N9 bonds are slightly lower than those for the N5‐N7/N6‐N9 bonds, consistent with the bond order analysis, which suggests that the N6‐N9 bond is marginally stronger. However, this minor difference does not significantly enhance the stability of *cyclo*‐N_6_. Additionally, the energy barrier for TS2 is notably low, indicating that IM1 is highly unstable. This finding is supported by Figure 4, where at 1000 K, the release of an N_2_ molecule is observed at step 750, followed by the formation of TS2 at step 835. The absence of a noticeable spike in RMSD suggests that these reactions occur almost simultaneously, over a very short timescale. For *cyclo*‐N_5_, the initial reaction energy barriers are ≈35 kcal mol^−1^, indicating relatively stable chemical bonds. This stability explains why no ring‐opening reactions were observed in the AIMD simulations. Based on the above analysis, it can be inferred that if *cyclo*‐N_9_^−^ undergoes self‐decomposition, the initial bond cleavage is likely to occur at *cyclo*‐N_6_, resulting in the product of the stable N_2_ molecules in the system.^[^ ^39^ ^]^ The remaining bonds have moderate bond orders, still indicating good stability.

### 2.2 Aromaticity Analysis

**Figure 6:**
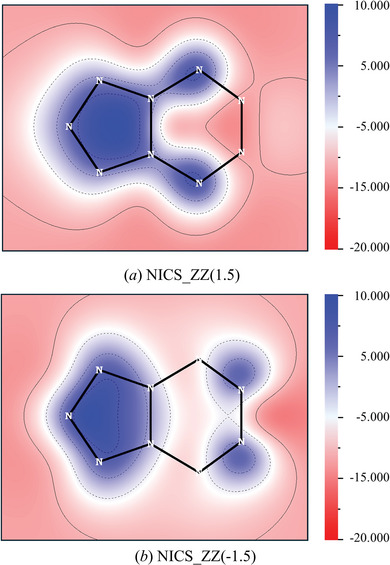
The 2D NICS_ZZ plane diagram at a distance of 1.5 Å from *cyclo*‐N_9_^−^, where a) represents the plane 1.5 Å above *cyclo*‐N_9_^−^ and b) represents the plane 1.5 Å below *cyclo*‐N_9_^−^.

C*yclo*‐N_9_^−^ consists of a fused ring structure, and aromaticity is crucial for the stability of rings. Therefore, investigating its aromaticity is essential. Currently, the most common method for determining aromaticity is Nucleus Independent Chemical Shift (NICS). It is based on the principle that the induced magnetic field from the ring current in a conjugated ring can counteract (shield) the external magnetic field to some extent. Conventionally, NICS_ZZ (1) is widely used, as it evaluates the aromaticity of a plane located 1 Å above the ring's center in the z‐direction, where σ‐orbital effects are minimized. However, NICS_ZZ (1) may not suitable for c*yclo*‐N_9_^−^, the distorted rings. In the case of c*yclo*‐N_9_^−^, the sub‐rings c*yclo*‐N_5_ and c*yclo*‐N_6_ are non‐coplanar, and c*yclo*‐N_6_ is particularly twisted. As a result, the standard NICS_ZZ (1) approach at 1 Å is insufficient to acquire the shielding tensor data and capture the aromaticity of c*yclo*‐N_6_ fully. Following the work of Dobrowolski et al.,^[^ ^40^ ^]^ this study uses the NICS_ZZ (±1.5) plane method to encompass the entire *cyclo*‐N_9_^−^. While this slightly reduces the π‐electron effect, it remains effective for qualitative aromaticity assessment. Two planes, each divided into a 200×200 grid, were used to comprehensively examine the aromaticity of *cyclo*‐N_9_^−^. The NICS_ZZ 2D plane at a distance of 1.5 Å is shown in **Figure** **6**.

From Figure 6a, it is evident that the left pentazole ring shows a deeper blue color, indicating that the electrons carried by each nitrogen atom in the ring are delocalized within the entire ring and create a shielding effect under the external magnetic field. The deeper the color, the stronger the magnetic shielding effect perpendicular to the system plane, confirming that the pentazole ring in *cyclo*‐N_9_^−^ is aromatic. However, the area enclosed by N5, N7, N8, and N9 shows minimal coloration. This absence of coloration is not due to the reaction of electrons under an external magnetic field but rather because the constructed plane is too distant from these atoms, rendering electronic effects invisible on the NICS_ZZ (1.5) plane. It should be noted that this limitation explains why NICS_ZZ (1) cannot fully validate the aromaticity of distorted rings and highlights the necessity of the NICS_ZZ (−1.5) plane. Figure 6b complements Figure 6a, and it can be observed that the hexazine ring also lacks blue regions, with blue areas mainly concentrated on and outside the ring. Combining the two figures, it is clear that the blue distribution areas on the hexazine ring are opposite to those on the pentazole ring, indicating antiaromaticity in the hexazine ring.

Additionally, iso‐chemical shielding surface (ICSS) calculations were performed to further verify the aromaticity of *cyclo*‐N_9_^−^. A total of 1 728 000 grid points were divided around the entire ion, and the resulting 3D chemical shielding surface with different magnetic shielding values is shown in Figure S2 (Supporting Information). Green represents shielding isosurface regions, while blue represents deshielding isosurface regions. With the magnetic induction ring current generated by π‐electrons, aromatic rings exhibit significant shielding inside the ring and deshielding regions around it in an external magnetic field, whereas antiaromatic rings show the opposite effect. When the shielding value is set to 0.50 ppm, a large green bulge appears in the middle of *cyclo*‐N_9_^−^, indicating overall aromaticity. However, when the shielding value is set to 3.00 ppm, the pentazole ring remains completely shielded, while the hexazine ring shows blue regions, indicating that the pentazole ring is aromatic, while the hexazine ring is antiaromatic. It is consistent with the NICS_ZZ analysis.

### 2.3 Topological Analysis of Electron Density

**Figure 7:**
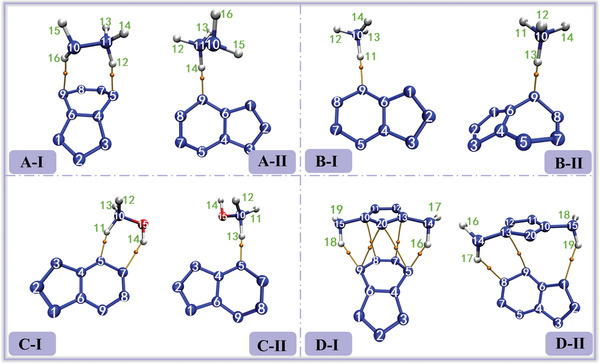
The topological analysis of electron density in the eight ionic systems, where yellow spheres represent the bond critical points.

Topological analysis of electron density distribution is a crucial method in the AIM theory for understanding molecular bonding. Bader defined points where the gradient of electron density is zero as critical points and classified them according to the curvature of electron density: 1) Nuclear critical points (NCP), 2) Bond critical points (BCP), 3) Ring critical points (RCP), and 4) Cage critical points (CCP).^[^ ^41^ ^]^ Generally, BCPs are located along the chemical bond path and are of particular interest as they reflect the interactions between two atoms. Therefore, less relevant critical points, such as nuclear critical points, are selectively omitted, while bond critical points are analyzed in detail. The topological images of electron density for the eight potential ionic systems are shown in **Figure** **7**, with the significant information summarized in **Table** **2**.

**Table 2:** The data from the topological analysis of electron density in the eight ionic systems.

System	Interaction	*ρ*[*r*]	▽^2^ *ρ*[*r*]	*H*[*r*]	*G*[*r*]	*V*[*r*]
A‐I	H16…N9	0.016	0.049	0.001	0.011	−0.009
	H12…N5	0.048	0.096	−0.009	0.033	−0.042
A‐II	H14…N9	0.059	0.095	−0.016	0.040	−0.056
B‐I	H11…N9	0.056	0.094	−0.014	0.038	−0.052
B‐II	H13…N9	0.049	0.094	−0.010	0.033	−0.043
C‐I	H11…N5	0.055	0.098	−0.013	0.038	−0.051
	H14…N7	0.027	0.089	0.001	0.021	−0.020
C‐II	N5…H13	0.075	0.080	−0.028	0.048	−0.075
D‐I	H16…N5	0.040	0.102	−0.004	0.030	−0.034
	N13…N7	0.011	0.046	0.002	0.010	−0.008
	N5…N20	0.013	0.042	0.001	0.010	−0.009
	N8…N10	0.011	0.046	0.002	0.010	−0.008
	N20…N9	0.013	0.042	0.001	0.010	−0.009
	N9…H18	0.040	0.102	−0.004	0.030	−0.034
D‐II	H17…N8	0.019	0.066	0.002	0.014	−0.012
	N9…N13	0.013	0.049	0.001	0.011	−0.009
	N1…H19	0.017	0.061	0.002	0.013	−0.011

At BCPs, the potential energy density (*V*) is typically negative, while the kinetic energy density (*G*) is usually positive, and the total energy density (*H*) is the sum of these two. Popelier suggested that the electron density at the BCP of a hydrogen bond falls within the range of 0.002–0.035 a.u.^[^ ^42^ ^]^ Rozas further proposed that if both the Laplacian of electron density (▽^2^ *ρ*(*r*)) and total energy density (*H*) are positive, the hydrogen bond is weak; if ▽^2^ *ρ*(*r*) is positive and *H* are negative, the hydrogen bond is of moderate strength; and if both are negative, the hydrogen bond is strong.^[^ ^43^ ^]^ From Table 2, it is evident that many H atoms form hydrogen bonds with highly electronegative atoms. For instance, in A‐I configuration, the hydrogen bond H16…N9 meets the criteria for a weak hydrogen bond. In C‐II, the only moderate hydrogen bond formed is N5…H13. Contrary to the judgment standards, many BCPs in the table fall outside the specified range of electron density values. However, this does not mean they are incorrect. In fact, higher electron density indicates stronger hydrogen bonds, which is a conclusion drawn from Lu's work.^[^ ^44^ ^]^ For example, according to the standard, the hydrogen bond H14…N7 in C‐I is relatively weak, whereas the electron density of H11…N5 exceeds the upper limit of the standard. As seen in Figure 7, the distance between H11 and N5 is shorter than that between H14 and N7, and no covalent bond is formed between them, indicating not only the presence of a hydrogen bond but also a stronger one. Moreover, most configuration I ionic compounds have more hydrogen bonds than configuration II, and these bonds are generally stronger, which is a significant reason for the greater stability of configuration I. However, it must be acknowledged that all hydrogen bonds play a vital role in stabilizing the entire system and enhancing the interactions between cations and anions.

### 2.4 IGMH Analysis

**Figure 8:**
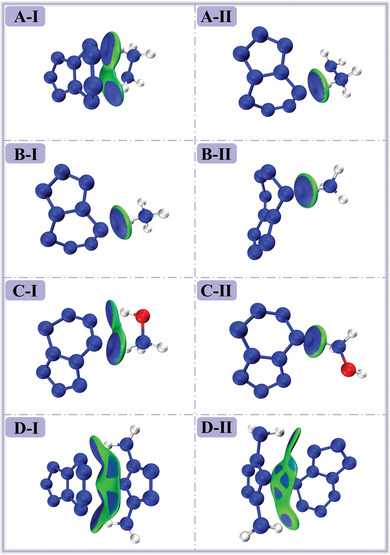
IGMH diagrams of the eight structures.

IGMH analysis is the most direct and effective way to understand intermolecular interactions through visualization. It not only identifies and characterizes the interaction regions between molecules but also evaluates the strength and nature of these interactions. To further understand the interactions between cations and anions in the eight ionic compounds, including [N_2_H_5_^+^] [N_9_^−^], **Figure** **8** shows IGMH diagrams of the eight structures. Different colors represent different types of interactions: red indicates repulsive interactions, green indicates van der Waals interactions, and blue indicates attractive interactions.

In all the configurations I on the left side, the number of hydrogen bonds is not less than that in the configurations on the right side, consistent with AIM analysis. Notably, in the A‐I structure, it is evident that although both the upper H12…N5 and lower H16…N9 regions exhibit blue coloration, indicating hydrogen bonds between the cation and anion, the upper region shows a more pronounced blue color, indicating higher electron density. This observation is corroborated by the data in Table 2. The electron density at the bond critical point for H12…N5 is 0.048, while for H16…N9, it is only 0.016. According to the standards set by Rozas and Lu,^[^ ^40^, ^41^ ^]^ the former hydrogen bond is stronger. While in the A‐II structure, the electron density between H14… N9 is 0.059, suggesting that the strength of the hydrogen bonds in A‐II is higher, although there are slightly more hydrogen bonds in A‐I. For [NH_4_^+^] [N_9_^−^], both configurations I and II show the formation of only one hydrogen bond between [NH_4_^+^] and [N_9_^−^]. However, due to the higher electron density in configurations I, the hydrogen bond is stronger, resulting in a more intense cation‐anion attraction and hence greater stability. Consequently, in most of the eight systems, the H atoms on the cation prefer to form hydrogen bonds with N9 or N5 atoms, leading to stronger attractive interactions. In the D‐II structure, an interesting phenomenon is observed: some light blue regions are distributed in a “petal” form on the interaction surface between the cation and anion, with only one nitrogen atom on *cyclo*‐N_9_^−^ located above the diamino‐pentazolium, as shown in Figure S3a (Supporting Information). Combining this with the charge analysis of *cyclo*‐N_9_^−^, it is evident that higher electron density results in LP‐π interactions between N9 atom and the aromatic *cyclo*‐N_5_, the view shown in Figure S3b (Supporting Information) strongly supports this point. In the configurations D‐I, Figure S4b (Supporting Information) illustrates that *cyclo*‐N_5_ overlaps with part of *cyclo*‐N_6_, forming π–π stacking interactions. Figure S4c (Supporting Information) displays the specific interaction regions between the two, while Figure S4a (Supporting Information) reveals that some π–π stacking interaction regions are connected with N‐H hydrogen bonds, making the interactions appear more complex. However, since the hexazine ring exhibits antiaromaticity, these interactions are weaker compared to the π–π stacking interactions between two aromatic rings. This is also evidenced by the lower electron density at the BCP of the N‐N interactions in AIM topological information.

### 2.5 Interaction Energy Calculation

For the four assembled nitrogen‐rich ionic systems, their interaction energies were calculated. When two fragments or groups interact, their basis functions overlap in the ionic system, which is equivalent to increasing the basis set of the system and reducing the E_(AB)_ energy. This problem is called the basis set superposition error (BSSE). Specifically, the “ghost atom” method was employed to perfectly address the BSSE,^[^ ^45^ ^]^ as shown in Equations (1),(2). Here, *E*_A_ and *E*_B_ represent the energies of the cation and anion monomers, respectively, while *E*_A,AB_ and *E*_B,AB_ are the energy of the monomers with the presence of ghost atoms. The interaction energies obtained from ORCA calculations and SAPT are presented in **Table** **3**.

(1) $$\Delta E_{interation}=E_{AB}-E_{A}-E_{B}+E_{BSSE}$$

(2) $$E_{\mathrm{BSSE}}=\left[E_{A}-E_{A,\mathrm{AB}}\right]+\left[E_{B}-E_{B,\mathrm{AB}}\right]$$

**Table 3:** Interaction energy data calculated by different methods.

Configuration	*E*_A,AB_	*E*_B,AB_	*E*_BSSE_	*E*_int,orca_	*E*_int,SAPT_
A‐I	−1291833.43	−294326.48	0.24	−449.58	−452.41
A‐II	−1291832.70	−294325.30	0.04	−440.71	−441.67
B‐I	−1291832.94	−149242.05	0.04	−442.56	−443.09
B‐II	−1291832.85	−149243.39	0.02	−430.07	−432.68
C‐I	−1291831.81	−346388.58	0.34	−504.36	−507.03
C‐II	−1291831.94	−346386.87	0.06	−459.89	−461.38
D‐I	−1291830.18	−1010395.38	0.58	−473.98	−483.03
D‐II	−1291833.80	−1010410.11	0.23	−424.70	−425.34

The unit of all energies is kJ mol^−1^.

**Figure 9:**
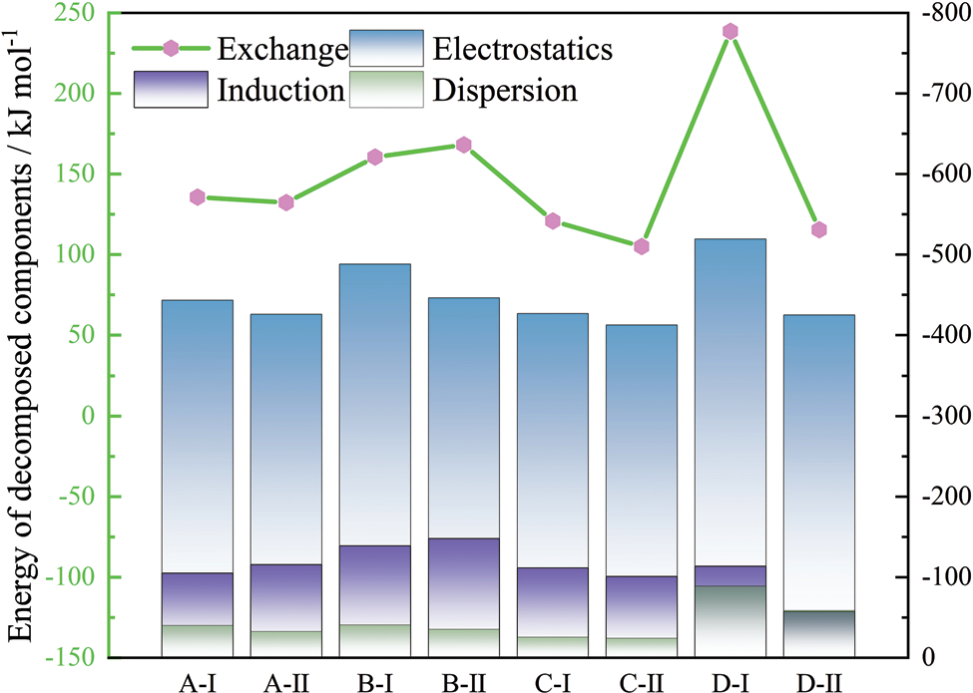
The energy decomposition details of each system.

Overall, configuration I of [NH_3_OH^+^] exhibits the smallest interaction energy and is the most stable structure, likely due to the strongest attractive forces from hydrogen bonds within [NH_3_OH^+^]. To further understand the components of the interaction energy, the SAPT method was used for a detailed decomposition, with all energy components illustrated in **Figure** **9**. Thanks to hydrogen bonds and π–π stacking interactions, the electrostatic interaction in configuration I of [N_7_H_4_^+^] is the highest, with a value of −519.178 kJ mol^−1^. However, due to the larger volume and suboptimal spatial arrangement of the cation, there are noticeable steric effects between the cation and anion, reflected in the exchange‐repulsion term, which hinders their binding. Additionally, the larger stacking area results in stronger van der Waals interactions between *cyclo*‐N_9_^−^ and [N_7_H_4_^+^], making dispersion interactions more significant in these two systems. Furthermore, the stronger polarity of the [NH_3_OH^+^] cation enhances the induction interaction between the cation and *cyclo*‐N_9_^−^ anion due to electron transfer between fragments. The smaller volume of [NH_3_OH^+^] also results in less pronounced exchange‐repulsion effects. Consequently, it is not surprising that configuration I of [NH_3_OH^+^] exhibits the smallest interaction energy and the most stable structure.

### 2.6 Electrostatic Potential Analysis

Electrostatic potential is essential for understanding intermolecular interactions and reactive sites by describing the charge distribution within molecules. To analyze the changes in *cyclo*‐N_9_^−^ after assembling with four different cations into ionic compounds, Figure S5 (Supporting Information) extensively illustrates the van der Waals surfaces colored by electrostatic potential and the penetration between cations and anions in the optimized composite systems. This work adopts the isosurface of electron density at 0.001 a.u, following Bader's methodology, as the van der Waals surface.^[^ ^46^ ^]^ In the figures, red regions indicate positive potential, blue regions indicate negative potential, and the deeper the color, the greater the absolute value of the electrostatic potential. Local minima and maxima on the isosurface are represented by cyan and orange spheres, respectively.

In the van der Waals penetration maps of the eight ionic systems, although the position of *cyclo*‐N_9_^−^ varies, the distribution of extremum points remains similar. The majority of minima for *cyclo*‐N_9_^−^ are situated near the hexazine ring, exhibiting comparable values that suggest elevated electron density in these regions. This makes them ideal targets for electrophilic reagents. Additionally, the charge distribution of cations in different isomers is also similar. Upon forming the composite systems, significant changes in electron distribution occur. The electron density above *cyclo*‐N_9_^−^ decreases markedly, and the minima observed in the isolated *cyclo*‐N_9_^−^ disappear in the composite systems. This is mainly due to electron migration to the regions where cations and anions penetrate each other, contributing to their binding. Due to differences in electronegativity, the maxima in the composite systems always appear near hydrogen atoms, while local minima remain near the N5 atoms. For example, in B‐I, the maxima near the hydrogen atoms of the cation is ≈100 kcal mol^−1^, while the minimum is −66.73 kcal mol^−1^, located to the right of the N5 atom. This indicates that, due to the unique molecular structure of *cyclo*‐N_9_^−^, electrons tend to accumulate in certain areas of the ring, increasing electron density even after pairing with cations. The uneven electron distribution also explains the antiaromaticity of the hexazine ring. However, it must be acknowledged that the combination of cations and anions inevitably reduces the polarity of the individual monomers. Compared to the extremum points in the van der Waals penetration maps, the absolute values of all extremum points in the ionic systems decrease to varying degrees, enhancing the overall chemical stability of the system and reducing the reactivity of the sites. Notably, in D‐I, the absolute values of most extremum points are significantly smaller. On one hand, the interaction region between *cyclo*‐N_9_^−^ and [N_7_H_4_^+^] is broader and stronger, requiring more electrons in the ionic bonding region. This leads to a reduction in electron density within the anionic region. On the other hand, the [N_7_H_4_^+^] has a wider electron distribution region that contains more electrons, resulting in lower inherent polarity. Consequently, the positive electrostatic potential on its surface is relatively smaller.

### 2.7 Detonation Performance

Detonation performance is a crucial metric for evaluating the excellence of any energetic material, and it is the primary reason for pursuing high nitrogen content. In this study, although the isomers of different systems contain the same types and numbers of atoms, their configurations can lead to variations in volume and density. Therefore, following Rice's suggestion,^[^ ^47^ ^]^ the corrected densities of the eight systems were calculated as shown in Equations (3)–(5), where $V_{s^{+}}$ and $V_{s^{-}}$ represent the electrostatic potential value of the cation and anion monomers, respectively.^[^ ^48^, ^49^ ^]^

(3) $$V_{\mathrm{uncorrected}}=pV_{M^{+}}+qV_{X^{-}}$$

(4) $$V_{\mathrm{corrected}\;}=V_{\mathrm{uncorrected}}\;-\;[0.6763+0.9418N]$$

(5) $$\rho (\mathrm{crystal})=\alpha (M/V_{\mathrm{corrected}})+\beta (V_{s^{+}}/A_{s^{+}})+\gamma (V_{s^{-}}/A_{s^{-}})+\delta$$

Enthalpy of formation is also a key factor in determining detonation performance. The calculation of enthalpy of formation for ionic compounds has its unique methods, as shown in Equations (6)–(8). Jenkins demonstrated that the actual enthalpy of formation of an ionic system is the sum of the formation enthalpies of the individual cation and anion, minus the lattice enthalpy.^[^ ^50^ ^]^ The primary factor affecting the lattice enthalpy is the lattice energy, with *γ* and *δ* being fitted empirical constants valued at 1981.2 mol^−^¹ cm and 103.8 mol^−^¹, respectively, which are applicable to M₁X₁‐type ionic compounds.^[^ ^51^ ^]^ The detonation performance of the eight systems was ultimately calculated using the K‐J equation and the EXPLO5 software package.^[^ ^52^, ^53^ ^]^ The K‐J equation is represented by Equations (9),(10). Here, *D* stands for detonation velocity, *P* represents detonation pressure, and *Q* denotes heat of detonation.

(6) $$\Delta H_{f}\left(\mathrm{salt},\;298\;K\right)=\Delta H_{f}\left(\mathrm{cation},\;298K\right)+\Delta H_{f}\left(\mathrm{anion},\;298K\right)-\Delta H_{L}$$

(7) $$\Delta H_{L}=U_{POT}+\left[p\left(n_{M}/2-2\right)+q\left(n_{X}/2-2\right)\right]RT$$

(8) $$U_{\mathrm{POT}}\left[\mathrm{kJ}\;\mathrm{mo}l^{-1}\right]=\gamma {\left(\rho_{m}/M_{m}\right)}^{1/3}+\delta$$

(9) $$D=1.01{\left(NM^{-1/2}Q^{1/2}\right)}^{1/2}\left(1+1.3\rho \right)$$

(10) $$P=1.558\rho^{2}N{\bar{M}}^{1/2}Q^{1/2}$$

**Figure 10:**
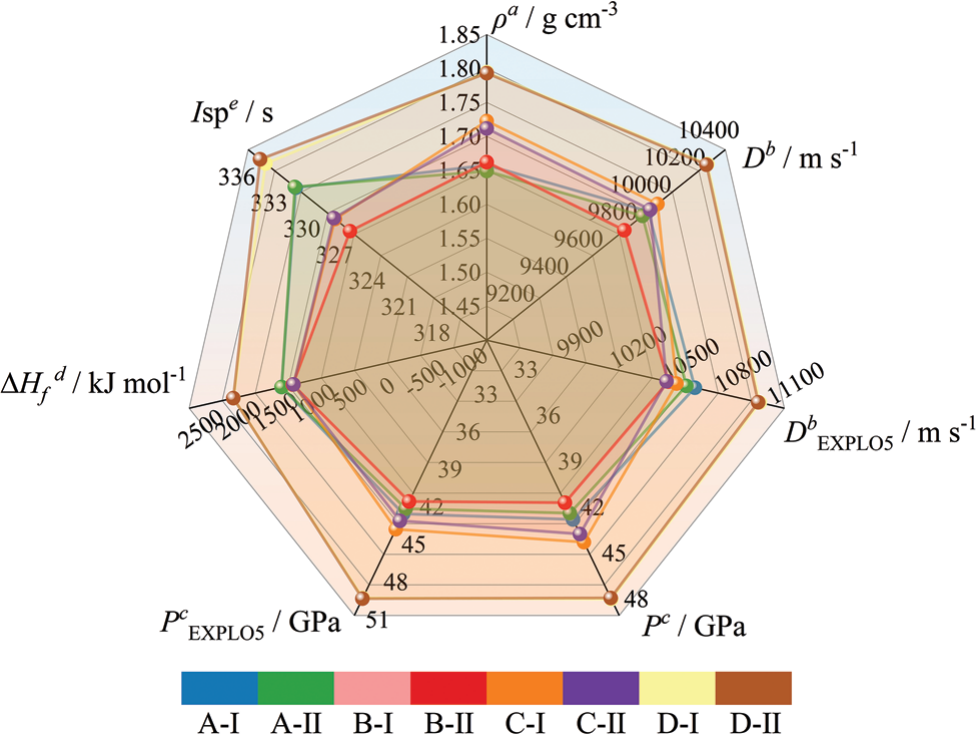
Detailed detonation performance parameters of eight systems. a) Calculated density. b) Detonation velocity, values calculated using the K‐J equation/EXPLO5 V6.05. c) Detonation pressure, values calculated using the K‐J equation/EXPLO5 V6.05. d) Calculated enthalpy of formation. e) Specific impulse of the neat compound using the EXPLO5 V 6.05 program package at 7 Mbar chamber pressure.

The crucial point that warrants first particular emphasis and clarification is that the statement that the energy of explosives is often inversely correlated with their stability and sensitivity doesn't align with the findings of this study. Configuration II demonstrates inferior stability, it also exhibits subpar performance. It's crucial to understand that the inverse relationship between energy and stability in energetic materials stems from the inherent instability of high‐energy chemical bonds. These bonds are more prone to breakage, making the entire system susceptible to decomposition. However, this principle doesn't apply to the isomers investigated in this study. Since they share the same atomic composition, the performance discrepancy between isomeric systems is primarily attributed to the varying interactions between their anions and cations. The stronger interactions result in a more tightly bound system, enhancing stability and density. Consequently, a seemingly positive correlation between detonation performance and stability emerges. It is essential to note that this observation differs fundamentally from the aforementioned energy‐stability relationships in general energetic materials. Besides, thanks to the extremely high enthalpy of formation, the data from the **Figure** **10** shows that the calculated detonation velocities for all systems using the K‐J equation exceed 9800 m s^−1^. Even more convincingly, the detonation velocities calculated by the EXPLO5 software are all above 10 500 m s^−1^. By comparison, the current most powerful energetic material, CL‐20, has a detonation velocity of only 9634 m s^−1^, making it significantly inferior to the *cyclo*‐N_9_^−^ ionic systems according to these calculations. Additionally, another nitrogen‐rich compound, C(N_5_)_4_, was shown by Zhang et al. in their work to have a maximum calculated detonation velocity of 10 300 m s^−1^ by using EXPLO5, which is evidently lower than any system in this study.^[^ ^54^ ^]^ More importantly, compared to their work, a lower number of nitrogen atoms (a maximum of only 16 nitrogen atoms) in these systems implies further improved stability and reduced synthesis difficulty. However, from the perspective of practical synthesis, [NH_3_OH^+^] [N_9_^−^] would be the foremost candidate for application in modern warfare. Its better stability not only increases its decomposition temperature but also significantly reduces its sensitivity. Although [NH_3_OH^+^] [N_9_^−^] exhibits somewhat inferior detonation performance compared to [N_7_H_4_^+^] [N_9_^−^], this slight performance loss is negligible in the face of substantially improved system stability. After all, the potential losses from accidental ignition and sympathetic detonation are far greater.

**Figure 11:**
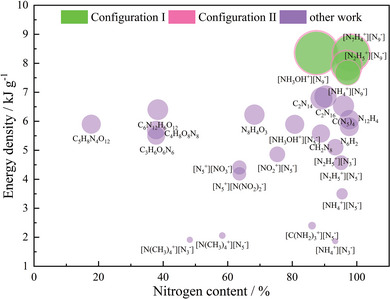
Comparison of energy densities among eight systems and other high‐energy materials.

Specifically, according to the suggestion of Zhang et al.,^[^ ^54^ ^]^ comparing the energy densities of the eight systems with other high‐energy compounds reveals their superior energy performance, as shown in **Figure** **11**. The data presented in the figure^[^ ^11^, ^54^, ^55^, ^56^, ^57^, ^58^ ^]^ leads to several distinct conclusions: the [N_7_H_4_^+^] [N_9_^−^], which exhibits the best detonation performance, also takes the lead in energy density. This can be primarily attributed to the presence of a higher number of high‐energy N─N bonds within the system. While [NH_3_OH^+^] [N_9_^−^] does not exhibit outstanding detonation performance, it surprisingly ranks highest in terms of energy density. This observation is thought‐provoking and warrants further investigation. First, it is noteworthy that the energy density of the system does not show a consistent trend of increasing with higher nitrogen content. The oxidation and heat release of hydrogen atoms by oxygen atoms in [NH_3_OH^+^] [N_9_^−^] system play a crucial role in its energy density, suggesting that improving the oxygen balance in nitrogen‐rich compounds may be an alternative effective approach to enhancing their energy performance. In addition, an increase in chemical bonds generally results in more energy release, this comes at the cost of adding more atoms. This leads to that even if some systems have lower energy densities, they still surpass those of other nitrogen‐rich compounds like C_2_N_14_ and C(N_5_)_4_, highlighting the superiority of the *cyclo*‐N_9_^−^ systems and demonstrating that the energy of the system is not entirely positively correlated with the nitrogen content. More importantly, it provides compelling evidence that [NH_3_OH^+^] [N_9_^−^] is indeed an excellent candidate for synthesis and practical application. These findings underscore the importance of a comprehensive evaluation of nitrogen‐rich compounds, considering not only the nitrogen content but also the roles of other elements and the overall chemical structure in determining their energy performance. Moreover, their favorable specific impulse values suggest that they could also play a significant role in propellant applications. It is worth noting that despite Politzer's efforts to account for factors affecting density, such as hydrogen bonds and intermolecular van der Waals interactions, did not realistically consider π‐π and LP‐π interactions between *cyclo*‐N_9_^−^ and other cations. As a result, the calculated densities of the eight systems are lower than their actual densities, which are expected to be more substantial. Therefore, the synthesis of these nitrogen‐rich compounds is highly anticipated.

## 3 Conclusion 

In this study, experimental work sparked our ideas, leading to the construction of *cyclo*‐N_9_^−^. The charge distribution in *cyclo*‐N_9_^−^ is not uniform, yet it exhibits a symmetrical pattern. The AIMD results at different temperatures are not only consistent with the bond order analysis but also reveal the primary decomposition pathways of *cyclo*‐N_9_^−^. Additionally, transition state calculations indicate that the stability of the chemical bonds on *cyclo*‐N_6_ is poorer, with the decomposition barrier being lower than that of *cyclo*‐N_5_. NICS and ICSS analyses reveal that electron delocalization in *cyclo*‐N_9_^−^ results in significant aromaticity in *cyclo*‐N_5_ and antiaromaticity in *cyclo*‐N_6_, while overall displaying aromaticity in the ring. Furthermore, in the eight ionic systems, hydrogen bonds occurs between *cyclo*‐N_9_^−^ and cations, with AIM characterizing the strength of hydrogen bonds and IGMH further determining their positions. Additionally, IGMH indicates the presence of LP‐π and π‐π stacking interactions between [N_7_H_4_^+^] and *cyclo*‐N_9_^−^, but the antiaromaticity of the hexazine in *cyclo*‐N_9_^−^ weakens the stacking interaction.

The interaction energy rankings of all compounds are C‐I<D‐I<C‐II<A‐I<B‐I<A‐II<B‐II<D‐II, indicating the highest stability of [NH_3_OH^+^] [N_9_^−^]. ESP analyses show lower polarity of reaction sites and higher chemical stability of [NH_3_OH^+^] [N_9_^−^]. In terms of explosive performance analysis, the energy density of all *cyclo*‐N_9_^−^ systems is notably significant compared to conventional energetic materials and other nitrogen‐rich compounds. Their detonation velocities exceed 9500 m s^−1^, with [N_7_H_4_^+^] [N_9_^−^] reaching an astonishing 11 000 m s^−1^. However, after comprehensive analysis, [NH_3_OH^+^] [N_9_^−^] is considered the most outstanding. Despite the slow progress in the research of polynitrogen and nitrogen‐rich compounds, this work once again demonstrates their bright prospects. It's important to have faith in the development of polynitrogen compounds, especially when we reflect on how CL‐20, renowned for its cage‐like structure, was once deemed a mere fantasy in the previous century.

## 4 Calculation Details

Unless otherwise specified, all geometry optimization and generation of intrinsic reaction coordinate (IRC) were performed using M062X‐D3/6‐311+G**,^[^ ^59^, ^60^, ^61^ ^]^ based on Gaussian 16, Revision A.03.^[^ ^62^ ^]^ The choice of M062X is primarily due to its suitability for conjugated systems and its superior accuracy in handling weak interactions compared to the commonly used B3LYP. Considering that *cyclo*‐N_9_^−^ and its composite compounds are ionic systems, the inclusion of polarization and diffuse functions is essential. The D3 dispersion correction, introduced by Grimme, further refines the M062X functional. Additionally, we optimized the structure of *cyclo*‐N_9_^−^ anion for many times by using the popular functional basis set combination ωB97XD/ma‐TZVP, B3LYP‐D3(BJ)/ma‐TZVP and the expensive but very accurate double hybrid functional revDSD‐PBEP86‐D3(BJ)/aug‐cc‐PVTZ.^[^ ^36^, ^37^, ^63^ ^]^ Although this behavior is quite extravagant, the results show that the *cyclo*‐N_9_^−^ structures obtained at these high precision levels are very consistent with those obtained by M062X‐D3/6‐311+G**, which ensures the reliability of the *cyclo*‐N_9_^−^ model and the applicability of the method in this work.^[^ ^64^ ^]^ Based on the obtained wave function information, the atomic charge distribution and bond order in *cyclo*‐N_9_^−^ were analyzed. An innovative approach allowed the NICS and ICSS theories to effectively validate its aromaticity.^[^ ^27^, ^65^, ^66^ ^]^ To further enhance the applicability of *cyclo*‐N_9_^−^, four cations—[N_2_H_5_^+^], [NH_4_^+^], [NH_3_OH^+^] and [N_7_H_4_^+^]—were assembled with *cyclo*‐N_9_^−^. During the screening and identification of the stable configurations of the four systems, the Molclus program was used to create 30 models for each ionic compound, totaling 120 models.^[^ ^67^ ^]^ Subsequently, two configurations were selected for each ionic system.^[^ ^68^, ^69^ ^]^ To improve the accuracy of energy calculations, single‐point energies and interaction energies for different configurations were computed at the DLPNO‐CCSD(T)/CBS with tightPNO and RIJK (ma‐def2‐TZVPP to QZVPP extrapolation) level, with all basis sets for energy extrapolation minimally augmented with diffuse functions.^[^ ^70^, ^71^, ^72^, ^73^, ^74^ ^]^ These calculations were performed using the ORCA 5.0.4 software package.^[^ ^75^ ^]^ The single‐point energy calculations for the transition state configurations follow the same approach, and the corrected Gibbs free energy was obtained through Shermo software package.^[^ ^76^ ^]^ The PSI4 software package^[^ ^77^ ^]^ was used to perform SAPT energy decomposition calculations for the eight systems at the “gold standard” SAPT2+(3)δMP2/aug‐cc‐pVTZ level,^[^ ^78^ ^]^ providing a comprehensive insight into the interactions between cations and anions. Further analysis of the interaction between cations and anions was conducted using AIM, IGMH, and ESP analyses to assess the stability of the ionic systems. Since the ultimate goal of designing and synthesizing energetic materials is practical application, the detonation performance of the four compounds was calculated. Notably, all the more precise thermodynamic data were calculated using the G4MP2‐6X composite method.^[^ ^79^ ^]^ The AIMD calculations at different temperatures were also performed using ORCA 5.0.4 software package, with a time step of 0.5 fs and a total duration of 20 ps. Atomic coordinate information was statistically analyzed at each step, and the entire process was conducted under the B3LYP/ma‐TZVP. The RMSD presented only shows the variations within the first 3000 steps of the dynamics, in order to better illustrate the initial decomposition process of *cyclo*‐N_9_^−^. Analysis processes were facilitated by the Multiwfn program^[^ ^80^, ^81^ ^]^ proposed by Lu and visualized using the VMD software package.^[^ ^82^ ^]^

## Conflict of Interest

The authors declare no conflict of interest.

## Author Contributions

Y.X. and M.L. conceived and designed the study. X.Y., Z.X., H.G. and T.Z. performed simulation calculations and wrote the paper. X.Y., Y.X. and M.L. reviewed and edited the manuscript. All authors read and approved the manuscript.

## Supporting information



Supporting Information

## Data Availability

The data that support the findings of this study are available in the supplementary material of this article.
