# Supplementary material for: Cyclo‐N9 −: a Novel 5/6 Fused Polynitrogen Anion for High Energy Density Materials
Source: Adv Sci (Weinh). 2025 Jan 28;12(19):2414394. doi: 10.1002/advs.202414394 (PMC12097120; doi:10.1002/advs.202414394)
Supplement: Supplementary file 1 — Supporting Information [file ADVS-12-2414394-s001.docx]

Supporting Information

***Cyclo*-N_9_^−^: A Novel 5/6 Fused Polynitrogen Anion for High Energy Density Materials**

*Xiaofeng Yuan*, *Ze Xu*, *Haolin Gu, Tongwei Zhang, Yuangang Xu*,* *Ming Lu**

**Table S1.** Fuzzy bond order (FBO), Wiberg bond order (WBO), and Laplacian bond order (LBO) of *cyclo*-N_5_^−^ and *cyclo*-N_9_^−^.

| System | Bond | FBO | WBO | LBO | Length (Å) |
| --- | --- | --- | --- | --- | --- |
| *cyclo*-N_5_^−^ | N-N | 1.620 | 1.805 | 1.076 | 1.315 |
| *cyclo*-N_9_^−^ | N1-N2/ N2-N3 | 1.615 | 1.784 | 1.117 | 1.310 |
|  | N3-N4/ N1-N6 | 1.401 | 1.481 | 0.923 | 1.327 |
|  | N4-N6 | 1.269 | 1.310 | 0.940 | 1.323 |
|  | N4-N5/ N6-N9 | 1.402 | 1.471 | 0.774 | 1.337 |
|  | N5-N7/ N8-N9 | 1.238 | 1.265 | 0.470 | 1.450 |
|  | N7-N8 | 2.062 | 2.325 | 1.706 | 1.221 |


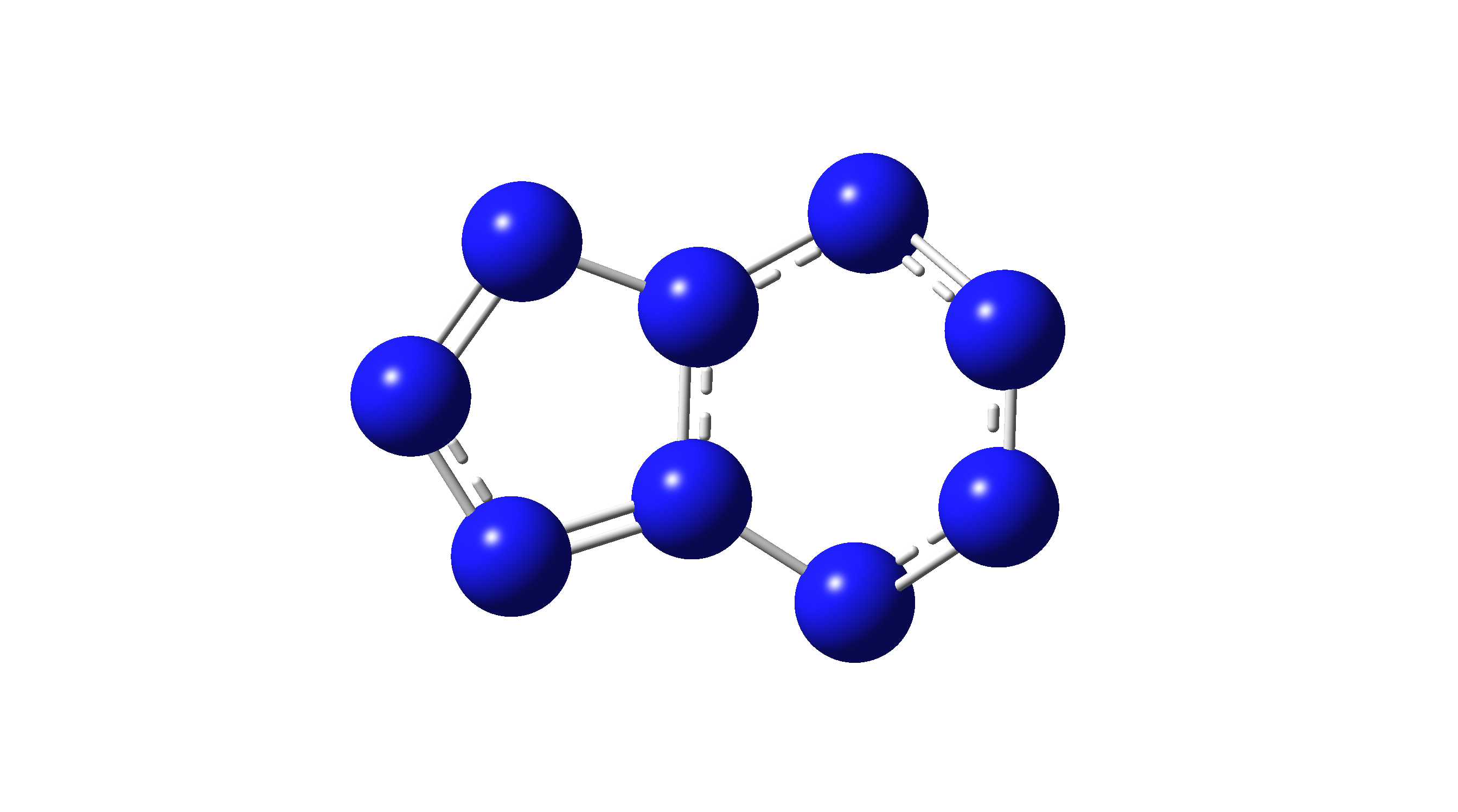


**Figure S1.** The optimized configuration of *cyclo*-N_9_^−^.





**Figure S2.** The three-dimensional diagram at different shielding values of ICSS analysis of *cyclo*-N_9_^−^.


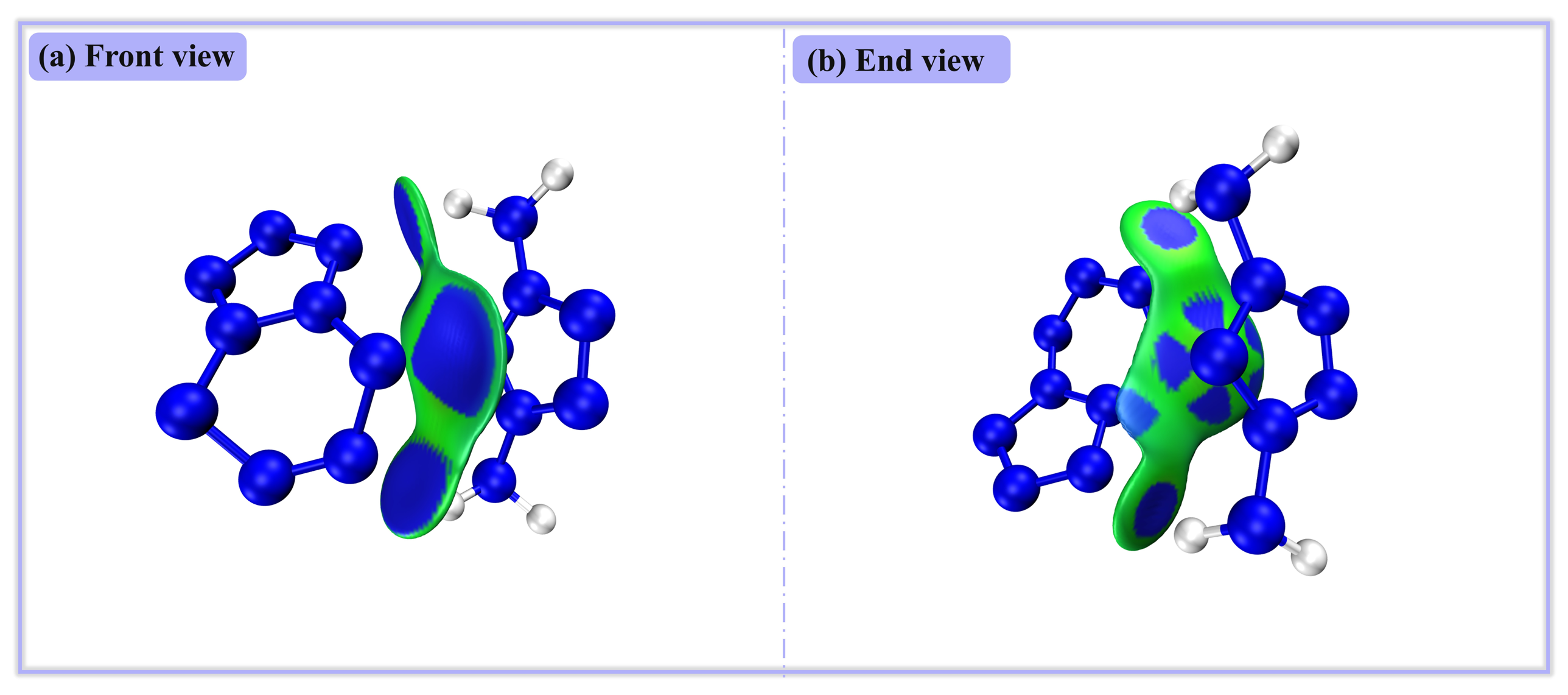


**Figure S3.** Visualization of D-Ⅱ structure from multiple perspectives


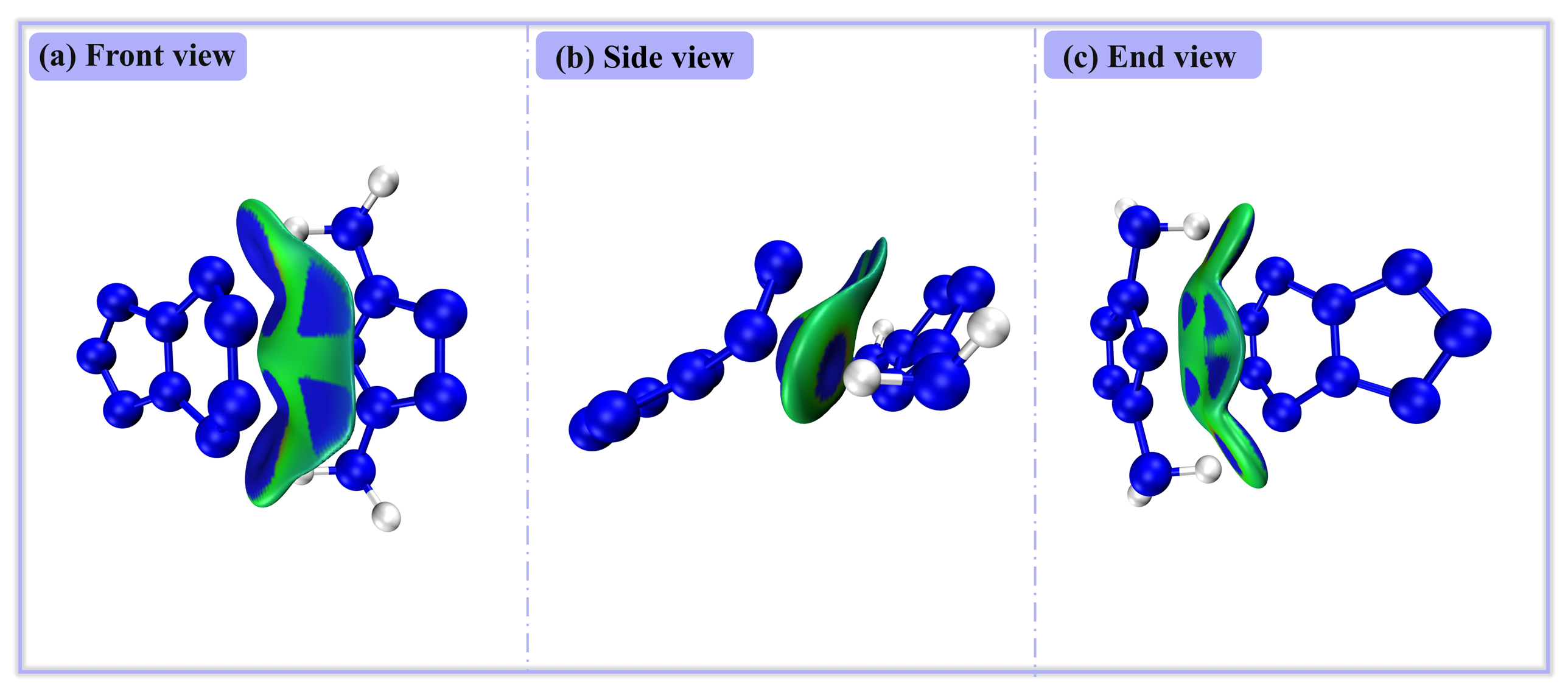


**Figure S4.** Visualization of D-Ⅰ structure from multiple perspectives








**Figure S5.** The electrostatic potential surface of the ionic systems composed of *cyclo*-N_9_^−^.
